# Conjugation of a Cationic Cell-Penetrating Peptide with a Novel Kunitzin-like Trypsin Inhibitor: New Insights for Enhancement of Peptide Bioactivities

**DOI:** 10.3390/pharmaceutics14091805

**Published:** 2022-08-27

**Authors:** Junting Yao, Weining Yin, Yuqing Chen, Xiaoling Chen, Yangyang Jiang, Tao Wang, Chengbang Ma, Mei Zhou, Tianbao Chen, Chris Shaw, Lei Wang

**Affiliations:** 1 School of Pharmacy, Queen’s University Belfast, 97 Lisburn Road, Belfast BT9 7BL, UK; 2 Department of Pharmacy, Affiliated Hospital of Nantong University, Nantong 226001, China

**Keywords:** cationic cell-penetrating peptides, Kunitz-type trypsin inhibitors, antibacterial activity, cancer cell proliferative activity

## Abstract

Cationic cell-penetrating peptides (CPPs), such as transactivator of transcription (TAT) peptide, have been proposed as effective drug carriers to improve intracellular delivery of biological macromolecules. Amphibian skin-derived Kunitz-type trypsin inhibitors (KTIs), short counterparts of KTIs from plant sources, were found to possess potent serine protease inhibitory activity. However, poor transmembrane permeability of these molecules has largely hindered the study of the full spectrum of their biological actions. As a result, this study aimed to extend the biological activities of amphibian KTIs by their conjugation to cationic CPPs. Herein, a novel peptide (kunitzin-OV_2_) and its phenylalanine-substituted analogue F9-kunitzin-OV_2_ (F9-KOV_2_) were evaluated for inhibition of trypsin/chymotrypsin and showed weak antibacterial activity against *Escherichia coli (E. coli)*. As expected, the conjugation to TAT peptide did not increase membrane lysis compared with the original kunitzin-OV_2_, but effectively assisted this complex to enter cells. TAT-kunitzin-OV_2_ (TAT-KOV_2_) exhibited a 32-fold increase in antibacterial activity and an enhanced bactericidal rate against *E. coli*. In addition, the conjugation enabled the parent peptides to exhibit antiproliferative activity against cancer cells. Interestingly, TAT-F9-kunitzin-OV_2_ (TAT-F9-KOV_2_) showed stronger antiproliferative activity against human breast cancer (MCF-7) and human glioblastoma (U251MG) cell lines, which TAT-KOV_2_ did not possess. Moreover, TAT-F9-KOV_2_ showed a 20–25-fold increase in antiproliferative capacity against human lung cancer (H157, H460) cell lines compared with TAT-KOV_2_. Therefore, the conjugation of CPPs effectively solves the problem of cell penetration that short KTIs lack and provides evidence for new potential applications for their subsequent development as new antibacterial and anticancer agents.

## 1. Introduction

Serine protease inhibitors widely exist in nature and play an essential role in maintaining homeostasis in many organisms, including humans [1]. As regulators, serine protease inhibitors participate in the immune response organisms and have been described to have clinically-significant roles in many diseases, such as inflammation, thrombosis, and cancer [2,3]. Among these inhibitors, Kunitz-type trypsin inhibitors (KTIs) are one of the most representative and popular groups in published research. The prototype was first isolated and crystallized from soybeans in 1945 and has one reactive center, which specifically inhibits trypsin [4]. KTIs are now known to be a large family, found in both animals and plants. Plant-derived KTIs have been extensively studied due to their multiple biological properties [5]. These include anti-pest functions through inhibiting insect protease activities and impeding insect digestion of proteins [6], prevention of cancer by inhibiting the amplification of mitogen-activated protein kinase (MAPK)-dependent urokinase signaling cascades [7], and inhibition of anti-inflammatory factor expression by inhibiting MAPK-dependent signaling pathways [8], etc.

Due to the extensive multidisciplinary research on this group of protease inhibitors, KTIs were found in amphibian skin secretions and were isolated and investigated. An amphibian-derived KTI was initially discovered in *Rana areolata*, a frog from North America [9]. However, the currently known amphibian-derived KTIs showed limitations on their bioactivity regulation and only exhibited inhibitory ability on several proteases, but had no effective in vitro antibacterial activities [10]. In general, unlike plant-derived KTIs with significantly longer sequences, amphibian-derived KTIs usually consist of less than 20 amino acids. These KTIs have retained the disulfide bond and the reactive center to maintain their protease inhibitory activity, but have lost the C-terminal α-helix structure in plant KTIs, explaining their inability to penetrate cells [4,10,11].

Cell-penetrating peptides (CPPs) are short peptides consisting of 5–30 amino acids, which can enter cells by carrying different bioactive substances specifically and non-specifically [12,13,14]. Cationic CPPs are mainly composed of abundant arginine or lysine residues with at least five positive charges, and their powerful delivery capacity for substances lacking membrane penetration ability has been experimentally confirmed [13,15]. In general, CPPs can enter cells in three ways: Direct membrane penetration, endocytosis mediated internalization, and formation of transmembrane transduction structures, but their exact transduction mechanisms are not well defined [16,17]. In the previous century, researchers discovered a transactivator of transcription (TAT) protein in the human immunodeficiency virus (HIV) and identified the so-called TAT sequence (YGRKKRRQRRR) with the ability to transport substances into cells [18,19,20]. Since then, a series of cationic CPPs with similar structures to TAT, such as polyarginine [21], low molecular weight protamine [22], and penetratin [23], have been widely used in inflammatory conditions as well as in cancer therapy studies [14].

Here, a novel peptide, named kunitzin-OV_2_, was discovered in the skin secretions of the Eastern Asian frog, *Odorrana versabilis*. It exhibited significant trypsin inhibitory activity as well as weak antibacterial activity. The analogue generated using phenylalanine to substitute the lysine at the P1 site of kunitzin-OV_2_ exhibited the inhibition of chymotrypsin. Next, TAT was conjugated to a synthetic replicate of the natural peptide and its synthetic phenylalanine-substituted analogues and the bioactivities of these peptides were evaluated. It was found that the antibacterial activity of the conjugates against *Escherichia coli* (*E. coli*) and the anticancer cell activity were significantly enhanced compared with the natural peptide. In addition, the conjugation of TAT to these peptides improved the in vitro bioactivities of the natural peptide and yet maintained the advantage of low eukaryotic cytotoxicity.

## 2. Materials and Methods

### 2.1. Harvesting of Odorrana Versabilis Skin Secretion

*Odorrana versabilis*, a species of frog in the family Ranidae that is endemic to China, is known simply as an odorous frog due to its foul-smelling feature. Previous research has revealed the extreme diversity of antimicrobial peptides (AMPs) in odorous frogs [24]. In addition to AMPs, protease inhibitors are generally found in amphibian skins. However, relatively little attention has been paid to the protease inhibitors from these “odorous” frogs, suggesting that this species has great potential to identify unknown protease inhibitors. Specimens of *Odorrana versabilis* (n = 6) were captured in Hainan, China. Cutaneous secretions of frogs were obtained from dorsal skin by electrical stimulation (5 V, 100 Hz, 140 ms pulse width). Thereafter, the white skin secretions obtained were washed from the dorsal skin using deionized water and collected in a 50-mL tube, frozen in liquid nitrogen, and placed in the freeze-dryer (Alpha, Germany) for lyophilization. The skin secretions after lyophilization were stored at −20 °C.

### 2.2. Identification of Precursor-Encoding cDNA from the Skin Secretion-Derived cDNA Library

To obtain biosynthetic peptide precursor-encoding cDNAs, a Dynabeads^®^ mRNA DIRECT^TM^ Kit (Biotech, Merseyside, UK) was used in a “shotgun” cloning experiment, in which Dynabeads oligo(dT) 25 (DBT) were used to isolate poly-A mRNA from lyophilized skin secretions of *Odorrana versabilis*. A SMART^TM^ RACE cDNA Amplification Kit (BD Biosciences Clontech, Basingstoke, UK) was used for cDNA library construction. The 3′-RACE PCR was used to obtain the nucleic acid sequence of the precursor peptide to full length. In the 3′-RACE experiment, a nested universal primer (NUP) within the kit and a degenerate sense primer (5′-GAWYYAYYHRAGCCYAAADATG-3′) were chosen as previously reported [25]. The RACE products were subjected to purification, molecular cloning, and sequenced using Cycle Pure Kit (Omega Bio-Tek, Norcross, GA, USA), pGEM-T vector system (Promega Corporation, Madison, WI, USA), and ABI 3100 automated sequencer (Applied Biosystems, Foster City, CA, USA), respectively. The resulting nucleotide sequence was analyzed in Expasy translate (https://web.expasy.org/translate/) (accessed on 5 November 2020) to obtain the open reading frame, and then analyzed in the NCBI-PROTEIN-BLAST (https://blast.ncbi.nlm.nih.gov/Blast.cgi/) (accessed on 5 November 2020)to predict the mature peptide sequence by contrasting sequence alignment with the precursors of bioactive peptides found in the skin of different species of amphibians in the past. This also identified the family of peptides to which the unknown belonged.

### 2.3. Peptide Design

There were two design strategies, both based on the parent peptide structure. The first was to target substitutions at the P1 site of the amphibian KTI. F9-kunitzin-OV_2_ (F9-KOV_2_) (AAKLPFRCFAAFC) is an analogue with chymotrypsin inhibition obtained by using phenylalanine to replace the lysine located at the P1 site of kunitzin-OV_2_ (AAKLPFRCKAAFC). In the second approach, to enhance the biological activity of kunitzin-OV_2_ and F9-KOV_2_, the classical short cationic CPP with cellular transport function, TAT_48–56_ (RKKRRQRRR) [26], was conjugated to kunitzin-OV_2_ and F9-KOV_2_ at the N-terminal and linked to them with a short linker (GG) to obtain the conjugates TAT-kunitzin-OV_2_ (TAT-KOV_2_) (RKKRRQRRRGGAAKLPFRCKAAFC) and TAT-F9-kunitzin-OV_2_ (TAT-F9-KOV_2_) (RKKRRQRRRGGAAKLPFRCFAAFC).

### 2.4. Solid Phase Peptide Synthesis (SPPS)

Kunitzin-OV_2_ and its three designed analogues were chemically-synthesized using the SPPS method. Briefly, the acid-sensitive polymer, Fmoc-Cys(Trt)-Wang (N-(9-fluorenylmethoxycarbonyl)-S-trityl-L-cysteine-Wang resin (Novabiochem, Darmstadt, Germany) was used as a carrier arm, while Fmoc was used as an α-amino protective group and the amino acids with side-chain functional groups were modified with the corresponding side-chain protective groups. For carboxyl activation, 2-(1H-benzotriazole-1-yl)-1,1,3,3-tetramethyluronium hexafluorophosphate (HBTU) and 11% 4-methylmorpholine (NMM) in dimethylformamide (DMF) were used in the C-terminal activation system. After the synthesis process in the Tribute^®^ Peptide Synthesiser (Protein Technology, Tucson, AZ, USA), the cleavage process was carried out using a mixture cleavage solution (trifluoroacetic acid (TFA)/deionized water/thioanisole (TIS)/1,2-ethanedithiol (EDT), 94/2/2/2, *v*/*v*/*v*/*v*) by placing it in a fume hood with constant stirring for 2 h. The cleavage solution was washed by diethyl ether (DEE), followed by centrifugal precipitation. The products were oxidized using hydrogen peroxide (H_2_O_2_/solution 0.2/99.8 *v*/*v*), then placed in the freeze-dryer (Alpha, Germany) for lyophilization over 48 h.

### 2.5. Purification and Identification of Kunitzin-OV_2_ and Its Analogues

A reversed-phase high-performance liquid chromatography (RP-HPLC) separation system was used to purify the crude peptides. The entire system was composed of an infusion pump (Adept CECIL CE4100), degasser (Adept CECIL CE4020), ultraviolet detector (Adept CECIL CE4200), column (Jupiter 5 µm Peptide Xb-C18 Column 250 × 21.2 mm), and Power Stream chromatographic workstation. The molecular masses of the peptides in HPLC fractions were detected by matrix-assisted laser desorption ionization, time-of-flight mass spectrometry (MALDI-TOF MS) (Voyager DE, Applied Bio-systems, Framingham, MA, USA), and 10 mg/mL α-cyano-4-hydroxy-cinnamic acid (CHCA) was used as the matrix. The sample target was placed into the mass spectrometer for analysis to obtain MS spectra by accumulating 50 single scanning signals (the MALDI-TOF mass spectra (Figure S1) and HPLC chromatograms (Figure S2) of the pure peptides are shown in the Supplementary Document).

### 2.6. Trypsin and Chymotrypsin Inhibition Determinations

Phe-Pro-Arg-AMC (Bachem, Merseyside, UK) and succinyl-Ala-Ala-Pro-Phe-AMC (Bachem, Merseyside, UK) were used as substrates for trypsin and chymotrypsin inhibition determinations, respectively. Serial peptide dilutions (0.1–100 µM in phosphate buffered saline (PBS)) were incubated with 180 µL of substrate solution (50 µM in PBS) in a black 96-well plate under dark conditions for 10 min. Immediately prior to measurement, 10 µL of trypsin/chymotrypsin working solution (0.1 µM in PBS) was added to each well (final volume of 210 µL) in the plate. Subsequently, the fluorescence intensity was measured with the FLUO star OPTIMA plate reader (BMG Labtech, Ortenberg, Germany). The whole measurement process lasted for 30 min and was carried out at an emission wavelength of 460 nm and an excitation wavelength of 395 nm. The inhibition curves of trypsin and chymotrypsin were analyzed by non-linear regression using the Morrison equation in Prism 9 (Trypsin Km = 67.38 μM; Chymotrypsin Km = 17.15 μM; [S] = 42.86 μM; Et = 0.0020 μM).

### 2.7. Primary Antimicrobial Screening by Minimal Inhibitory Concentration (MIC) and Minimal Bactericidal Concentration (MBC) Assays

The antimicrobial efficacy screening of the purified peptides was performed by MIC/MBC experiments on the following six different bacteria and an opportunistic pathogenic yeast: *E. coli* (ATCC 8739), *Klebsiella pneumoniae* (*K. pneumoniae*) (ATCC 43816), *Pseudomonas aeruginosa* (*P. aeruginosa*) (ATCC 9027), *Staphylococcus aureus* (*S. aureus*) (ATCC 6538), methicillin-resistant *Staphylococcus aureus* (MRSA) (NCTC 12493), *Enterococcus faecium* (*E. faecium*) (NCTC 12697), and *Candida albicans* (*C. albicans*) (ATCC 10231). First, frozen beads of respective cultures were inoculated into Mueller Hinton broth (MHB) medium and incubated overnight at 37 °C. Next, 500 µL of respective suspensions were added to the fresh MHB medium for incubation, and then sub-cultured until reaching the log growth phase, which was determined by measuring the absorbance of cultures at a wavelength of 550 nm. Then, the subcultures were diluted to 5 × 10^5^ CFU/mL and 99 µL of suspension was removed and mixed with 1 µL of peptide solution at different concentrations (double-dilution of 12,800 to 100 µM) in a 96-well plate. The blank control, vehicle control, growth control, and the positive control groups were prepared according to the conditions mentioned previously [27]. The peptide solutions were added to the plate and mixed with the diluted microbial suspensions. After overnight incubation, the absorbance at 550 nm of each well was measured with a Synergy HT plate reader (Biotech, Minneapolis, MN, USA). Subsequently, the samples in each clear well were spotted onto Mueller Hinton agar (MHA) plates for the MBC assays of the tested peptides.

### 2.8. Antibiofilm Assays

To explore the ability of peptides to inhibit and eradicate the biofilm of *E. coli*, the minimum biofilm inhibition concentrations (MBICs) and minimum biofilm eradication concentrations (MBECs) were tested, respectively. Luria-Bertani broth (LB) medium containing 1% glucose was used to culture *E. coli*. The subcultured bacteria were diluted to 5 × 10^5^ CFU/mL and 99 µL of this bacterial suspension was used to mix with 1 µL of peptide solution at different concentrations (double-dilution of 12,800 to 100 µM) in a 96-well plate. After overnight culture in an oscillating incubator at 37 °C and 220 rpm, the formed biofilm was washed twice with 100 µL PBS, then fixed in methanol. Next, biofilm formation was evaluated by 0.1% crystal violet staining. After dyeing for 15 min, the excess crystal violet was washed off and 30% glacial acetic acid was added to each well of a 96-well plate after drying. Then, the plate was shaken for 15 min, and the absorbance was measured at 595 nm using the Synergy HT plate reader. For the determination of MBEC, after the biofilm was formed, it was first washed twice with PBS, then the fresh medium and different concentrations of peptide (similar to MBIC experiments) were added and incubated at 37 °C at 220 rpm. The absorbance measurement was the same as previously described after 24 h of MBIC.

### 2.9. Time-Killing Kinetics Assays

The time-killing kinetics assay is used to evaluate the killing kinetics of bacteria treated with peptides. *E. coli* was cultured in MHB medium as mentioned in the MIC experiment previously. The concentrations of 4 × MIC, 2 × MIC, and 1 × MIC peptide solutions, were inoculated with cultured bacteria. When the peptide had been added to the bacterial suspension, samples were removed at different time points (0, 5, 10, 20, 30, 60, 90, and 120 min)—20 μL samples of bacteria-peptide mixture were removed and added to 180 μL sterile PBS, diluted 10-fold, and then 6 additional 10-fold dilutions were prepared. Then, 10 μL of each dilution of bacteria/peptide sample at different concentrations were dropped onto an MHA plate for live cell counting. After incubation at 37 °C for 24 h, the number of colonies at different concentrations and time points was calculated.

### 2.10. Membrane Lysis Assays

Cell lysis was assessed on *E. coli* using the SYTOX^TM^ Green Nucleic Acid Stain (Life Technologies, UK). For *E. coli*, overnight bacterial suspensions were passaged in LB medium for 2.5 h. At the end of the passage, the cells were washed twice with 5% LB/0.85% NaCl (*v*/*v*) and the bacterial cells were resuspended with 5% LB to reach a concentration of 1 × 10^8^ CFU/mL. The absorbance at this concentration was measured at 590 nm (OD = 0.7). After completion of bacterial preparation, as the positive control group, 1 mL of bacterial culture was treated with 70% isopropanol for 1 h to allow complete penetration and rupture of the bacterial cell membrane. For the sample group, peptides were spiked at a final concentration of 1 × MIC, 2 × MIC, and 4 × MIC of peptides, in wells of a 96-well plate. The same volume of 5% LB substitute peptide solution was used as the negative control group. After incubation at 37 °C for 2 h, the stain was added to each well in dark conditions and shaken to mix well. Subsequently, the absorbance was measured using the Synergy HT plate reader at an excitation/emission wavelength of 485/528 nm.

### 2.11. Salt Sensitivity Assays

To determine the salt sensitivity of peptides, *E. coli* was used to detect the changes in MIC values of peptides under different salt conditions. The bacterial culture method used was similar to the MIC experimental method described earlier. Different concentrations of salts (150 mM NaCl, 4.5 mM KCl, 6 μM NH_4_Cl, 1 mM MgCl_2_, 1 mM MgCl_2_, 8 mM ZnCl_2_, 2.5 mM CaCl_2_, 4 mM FeCl_3_) were added to the MHB to examine the effects of each salt on the anti-*E. coli* activities of the peptides, as described in a previous study [28]. Then, the bacterial dilution with salt was mixed well with peptides of different concentrations, similar to the MIC/MBC experiments, and cultured at 37 °C. After 18–20 h of incubation, the absorbance of each well was measured at 550 nm using the Synergy HT plate reader.

### 2.12. MTT Cell Antiproliferation Assays

The antiproliferative activities of the synthesized peptides were assessed on six human cancer cell lines (PC-3, H157, H460, H838, MCF-7, U251MG) and two human normal cell lines (HMEC-1 and MRC-5) using the MTT cell viability assay. Human lung cancer cells (H838, H157, and H460) and human prostate cancer cells (PC-3) were cultured in complete growth medium (CGM) (Roswell Park Memorial Institute (RPMI) 1640 Medium (Gibco, Paisley, UK): Fetal bovine serum (FBS) (Gibco, Campinas, Brazil): Penicillin-streptomycin solution (PS) (Gibco, Grand Island, NY, USA) 89/10/1 *v*/*v*/*v*). Human glioblastoma astrocytoma (U251MG) and human breast cancer cell (MCF-7) were cultured in complete growth medium (Dulbecco’s Modified Eagle medium (DMEM) (Gibco, Paisley, UK): FBS: PS 89/10/1 *v*/*v*/*v*). Human microvascular endothelial cells (HMEC-1) and human pulmonary fibroblast cells (MRC-5) were cultured in MCDB131 medium (supplemented with 10% FBS, 1% PS, 10 ng/mL epidermal growth factor, 1 µg/mL hydrocortisone, and 10 mM glutamine) and Eagle’s Minimal Essential Medium (EMEM) (supplemented with 10% FBS and 1% PS), respectively. Cell suspension, containing 8 × 10^3^ cells per 100 µL, was first added to 96-well plates for overnight incubation. Then, the adherent cells in the 96-well plates were subject to cell starvation using the serum free medium treatment for at least 4 h to ensure that the cells were all in the same growth cycle. The peptide solutions, with final concentrations of 10^−9^–10^−4^ M, were prepared with serum free medium and added onto the plate. In addition, Triton X-100 (0.1%) (Sigma-Aldrich, St. Louis, MO, USA) was used as a positive control. After 24 h of administration, the MTT solution was added to the plate and incubated for 2 h. Then, the supernatant was discarded and DMSO was added to dissolve the purple formazan crystals. The absorbance was measured in all wells using the Synergy HT plate reader at a wavelength of 570 nm.

### 2.13. Haemolysis Activity Assays

Horse erythrocytes (TCS Biosciences Ltd., Buckingham, UK) were washed completely with PBS and resuspended to obtain a 2% horse erythrocyte suspension [29]. Then, the peptide solutions at different concentrations were gently mixed with the same volume of horse red blood cell suspension and the mixture of peptide and horse erythrocytes was placed into a humidified incubator at 37 °C for 2 h. After incubation, the mixture of all experimental groups was centrifuged at 900× *g* for 10 min and the supernatants obtained were transferred to a blank 96-well plate. The absorbance of each well was measured at 550 nm using the Synergy HT plate reader.

### 2.14. Lactate Dehydrogenase (LDH) Cytotoxicity Assays

A CyQUANT™ LDH Cytotoxicity Detection Kit (Thermo Fisher Scientific, Waltham, MA, USA) was used to detect the LDH released from human microvascular endothelial cell line (HMEC-1) and human pulmonary fibroblast cell line (MRC-5). Cells were cultured as for the MTT antiproliferation assay described previously. After incubation, the peptide solutions at the same concentrations as in the previous method were added to the cells for further incubation of 24 h. Among these, the positive control group, representing the maximal LDH release, was obtained from the 10 × lysis buffer supplied in the kit. The lysis buffer was added to the control group and co-cultured for 45 min, while deionized water was added to the spontaneous LDH release control. Subsequently, the same volume of sample as the reaction mixture was withdrawn, added into a new 96-well plate, and mixed well. After co-culturing under dark conditions for 30 min, an equal amount of stop solution was added to stop the reaction. Absorbance values of all experimental groups were measured at wavelengths of 490 and 680 nm using the Synergy HT plate reader.

### 2.15. Statistical Analysis

All the numerical data obtained were analyzed by the GraphPad Prism 9 software (GraphPad, San Diego, CA, USA). The error bars in all the graphs were obtained by calculating the standard error of the mean (SEM) for data from all the experimental groups.

## 3. Results

### 3.1. Peptide Synthesis

#### 3.1.1. “Shotgun” Cloning of Kunitzin-OV_2_ Precursor cDNA

**Figure 1:**
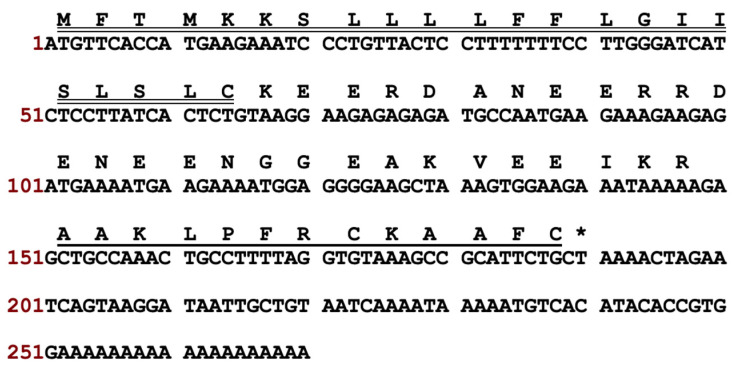
Nucleotide sequence and corresponding translated amino acid sequence of kunitzin-OV_2_ precursor cDNA. The putative signal and mature peptides are marked by double and single underlines, respectively. The stop codon is indicated by an asterisk.

**Figure 2:**
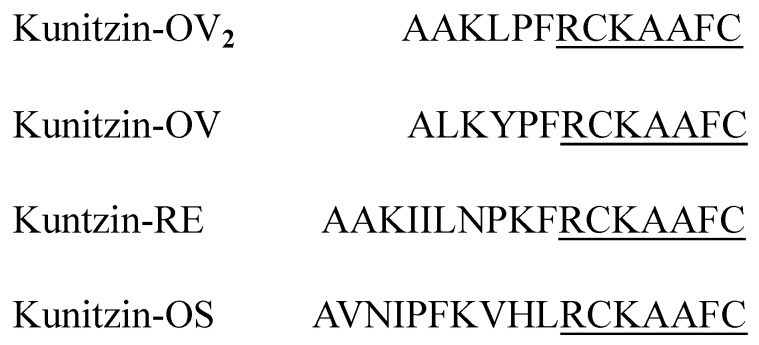
Mature peptide sequence of kunitzin-OV_2_ and previously published kunitzin peptides [10,11]. Fully conserved domains are single-underlined.

The full-length nucleotide sequence of cloned kunitzin-OV_2_ precursor cDNA was obtained by molecular cloning from a cDNA library constructed from the skin secretions of *Odorrana versabilis* (Figure 1). The putative signal peptide was separated from the mature peptide by an acidic amino acid-rich spacer peptide, ending in the cleavage site (-Lys-Arg-). The signal and mature peptides are marked by double and single underlines, respectively. By performing NCBI-BLAST analysis, the sequence of kunitzin-OV_2_ was found to exhibit a high similarity to a peptide from the kunitzin family reported previously (Figure 2). The nucleotide sequence of the kunitzin-OV_2_ precursor has been deposited in the GenBank database under accession number ON866881.

#### 3.1.2. Design of Short Peptides from the Parent Peptide Kunitzin-OV_2_

F9-KOV_2_ was designed based on kunitzin-OV_2_ by substituting lysine with phenylalanine at the P1 site. The aim was to produce a peptide with chymotrypsin inhibitory activity. The TAT peptide and linker (GG) were added at the N-terminus of the kunitzin-OV_2_ and F9-KOV_2_ to increase the membrane penetration ability. Moreover, the grand average of hydropathicity (GRAVY) values of peptides were calculated by Expasy-ProtParam (https://web.expasy.org/protparam/) (accessed on 3 July 2021) (Table 1).

### 3.2. Bioactivity Determination

#### 3.2.1. Trypsin/Chymotrypsin Inhibitory Effects

**Figure 3:**
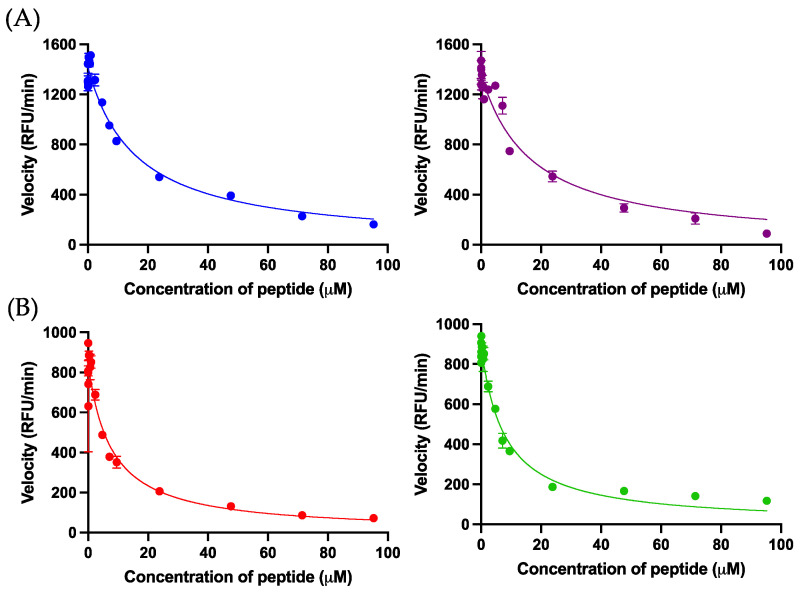
(**A**) The Morrison inhibition curves of kunitzin-OV_2_ (blue) and TAT-KOV_2_ (purple) against trypsin. (**B**) The Morrison inhibition curves of F9-KOV_2_ (red) and TAT-F9-KOV_2_ (green) against chymotrypsin. The X-axis represents peptide concentrations and the Y-axis represents the velocity of the fluorescence decay. The Ki values were calculated by Morrison formula in Prism 9, in which trypsin Km = 67.38 μM, chymotrypsin Km = 17.15 μM, [S] = 42.86 μM, Et = 0.0020 μM. The error bar represents the SEM of nine replications in three experiments.

Kunitzin-OV_2_ exhibited inhibition activity against trypsin and the substitution of phenylalanine in P1 position conferred chymotrypsin inhibition on F9-KOV_2_. As expected, the TAT-KOV_2_ and TAT-F9-KOV_2_ retained their trypsin and chymotrypsin inhibition activities, respectively (Table 2). The relevant Morrison inhibition plots are shown in Figure 3.

#### 3.2.2. Screening the Antimicrobial Effects of Kunitzin-OV_2_ and Its Analogues

The antimicrobial activity of test peptides was evaluated using a representative set of microorganisms. The results of MIC and MBC experiments are summarized in Table 3. It was found that the parent peptide, kunitzin-OV_2_, could only inhibit the growth of *E. coli* at the highest test concentration of 128 µM and its analogue F9-KOV_2_ did not retain the antimicrobial effect. As expected, the antibacterial abilities of the parent peptide conjugated with TAT against *E. coli* and *S. aureus* were significantly improved, especially for *E. coli* where the MIC was increased by around 32-fold (MIC = 4 µM). Moreover, TAT-F9-KOV_2_ exhibited a good antimicrobial effect against *E. coli* (MIC = 8 µM) and a moderate effect against *S. aureus* (MIC = 64 µM). In addition, both parent peptides and conjugates did not show any significant inhibitory activity against other tested microbes. These data suggested that the conjugation of a cationic CPP to kunitzin-OV_2_ greatly improves its anti-*E. coli* activity.

#### 3.2.3. Antibiofilm Activity

**Figure 4:**
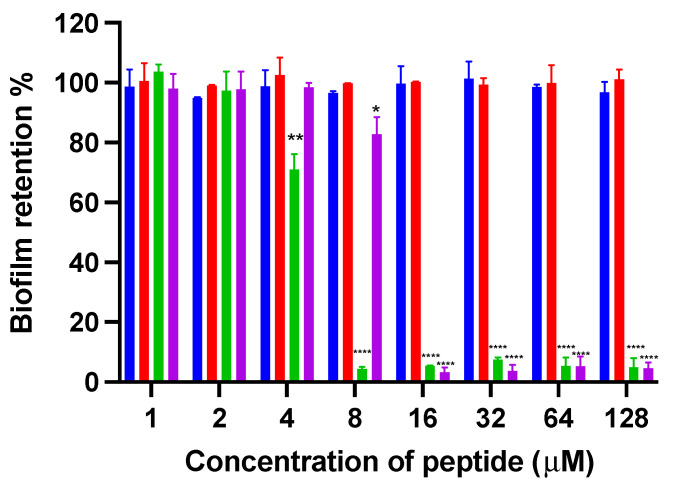
The inhibition of biofilm formation of kunitzin-OV_2_ (blue), F9-KOV_2_ (red), TAT-KOV_2_ (green), and TAT-F9-KOV_2_ (purple) against *E. coli* at the concentration from 1 to 128 µM. The bacterial treated with norfloxacin (20 mg/L) and sterile LB medium (1% glucose) were used as the positive and negative controls in the biofilm formation inhibition experiment, respectively. The error bar represents the SEM of nine replications in three experiments and the significant difference results were calculated by comparing various treatments with the negative control (* *p* < 0.05, ** *p* < 0.01, and **** *p* < 0.0001).

The antibiofilm activities of kunitzin-OV_2_, F9-KOV_2_, TAT-KOV_2_, and TAT-F9-KOV_2_ (Figure 4) were assessed. The parent peptides, kunitzin-OV_2_ and F9-KOV_2_, did not exhibit any inhibitory effect on biofilm formation at 128 µM. After conjugating with TAT, their antibiofilm capacities against Gram-negative bacteria were significantly improved. Compared with parent peptides, TAT-KOV_2_ and TAT-F9-KOV_2_ could strongly inhibit the biofilm formation of *E. coli* at 8 and 16 µM, but did not exhibit effects on the existing biofilms, even at the highest experimental concentration (128 µM). These data indicated that the small AMP, conjugated to a cationic CPP, might only act on free bacteria before biofilms are formed. Therefore, the data demonstrated the protective effects of biofilm once it is established.

#### 3.2.4. *E. coli*-Killing Kinetics

**Figure 5:**
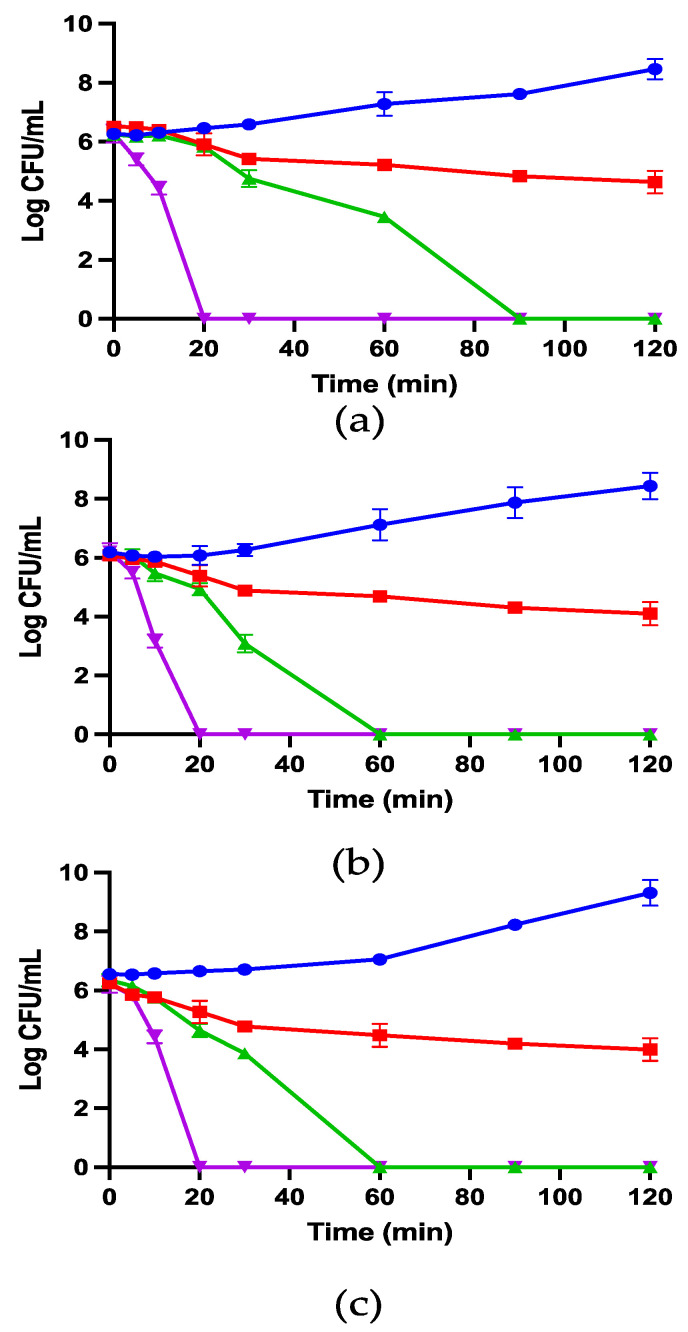
The kinetic time-killing curves of kunitzin-OV_2_ (**a**), TAT-KOV_2_ (**b**), and TAT-F9-KOV_2_ (**c**) against *E. coli* at the concentrations of 1/2 × MIC (red), 1 × MIC (green), 2 × MIC (purple), and the growth control (blue) with no peptide added. Y-axis represents log CFU/mL for all groups, which is determined at time 0 and at subsequent time points up to 120 min. The error bar represents the SEM of nine replications in three experiments.

In this study, 1/2 × MIC, 1 × MIC, and 2 × MIC concentrations of kunitzin-OV_2_, TAT-KOV_2_, and TAT-F9-KOV_2_ were employed to conduct time-killing experiments to explore the influence of TAT conjugation with cationic CPP on the bactericidal kinetics against *E. coli*. As shown in Figure 5, the peptide conjugated to TAT showed faster killing kinetics than the maternal peptide. Specifically, at MICs, TAT-KOV_2_ killed all bacteria within 60 min, whereas more than 1 × 10^3^ CFU/mL bacteria survived after 60 min of co-incubation with the kunitzin-OV_2_. For TAT-F9-KOV_2_, although it had a similar bactericidal speed as TAT-KOV_2_ at MICs, when they were all at the same concentration (4 µM), more than 1 × 10^4^ CFU/mL surviving bacteria were observed even after 120 min, while TAT-KOV_2_ killed all bacteria within 60 min. Furthermore, all tested peptides could kill all bacteria within 20 min at 2 × MICs. Therefore, it has been well demonstrated that TAT-KOV_2_ has potential as a more efficient bactericide.

#### 3.2.5. SYTOX Green Permeabilization

**Figure 6:**
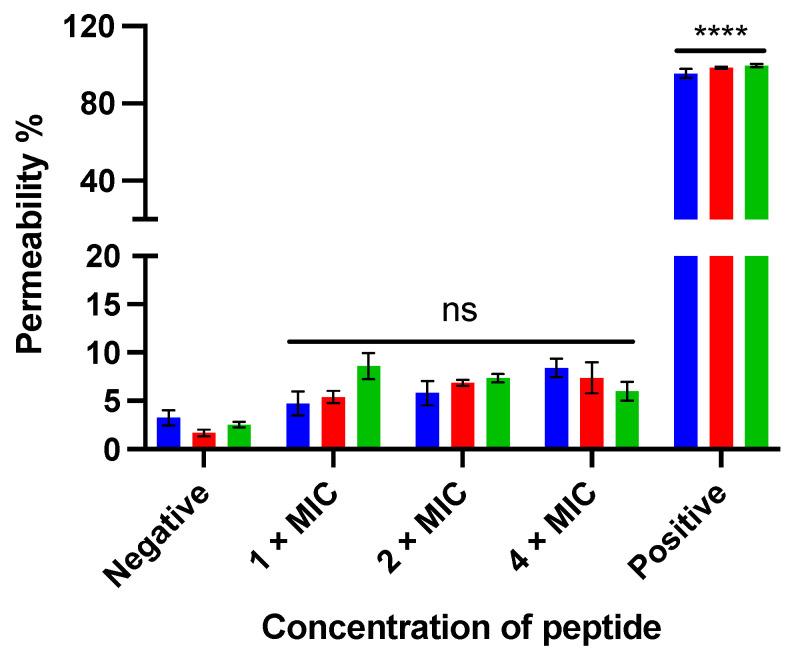
The membrane permeabilizing effect of kunitzin-OV_2_ (blue), TAT-KOV_2_ (red), and TAT-F9-KOV_2_ (green) against *E. coli* at the concentration of 1 × MIC, 2 × MIC, and 4 × MIC. The bacterial treated with 70% isopropanol and 0.85% NaCl solution (5% LB) was used as the positive and negative controls, respectively. The permeability percentage was obtained by comparing it to the fluorescent intensity of positive control. The error bar represents the SEM of nine replications in three experiments and the significant difference results were calculated by comparing various treatments with the negative control (**** *p* < 0.0001, ns: No significance).

The membrane permeabilizing effect of kunitzin-OV_2_ and its analogues against *E. coli* was analyzed by SYTOX green nucleic acid staining. As shown in Figure 6, after the peptide was incubated with bacteria for 120 min, the parent peptide and the conjugates showed no significant membrane damage even at the maximum test concentration of 4 × MIC. The results indicate that the antimicrobial effect of kunitzin-OV_2_, TAT-KOV_2_, and TAT-F9-KOV_2_ does not appear to be directed by membrane lysis, and they also suggest that the conjugation of a cationic CPP to the peptides does not affect cell membrane permeabilization.

#### 3.2.6. Salt Sensitivity

The sensitivity of the anti-*E. coli* activities of kunitzin-OV_2_ and its analogues to different salts was tested. The results are shown in Table 4. It was found that all tested salts reduced the anti-*E. coli* effect of kunitzin-OV_2_. However, the anti-*E. coli* activities of TAT-KOV_2_ and TAT-F9-KOV_2_ were shown to be sensitive only to MgCl_2_ and CaCl_2_ and their MICs improved by 2-fold and 4-fold, respectively.

#### 3.2.7. Anticancer Cell Proliferative Activity

**Figure 7:**
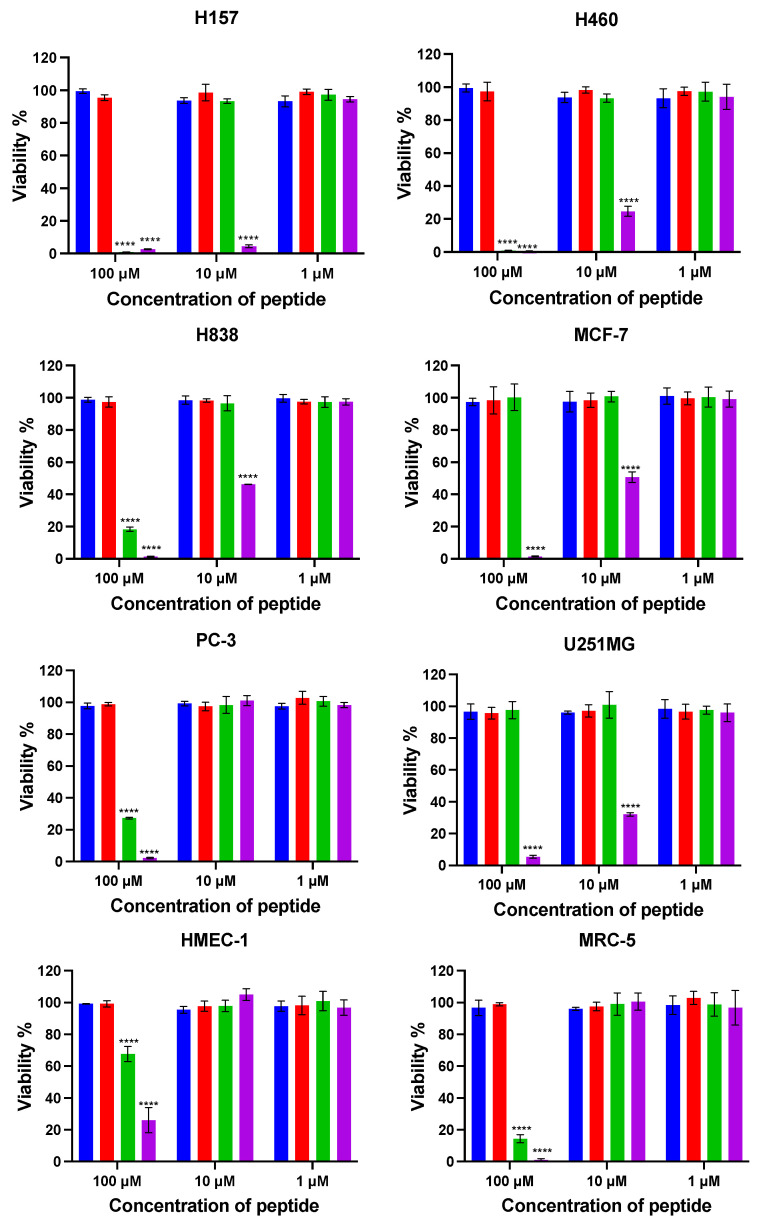
The cell viabilities were obtained after kunitzin-OV_2_ (blue), F9-KOV_2_ (red), TAT-KOV_2_ (green), and TAT-F9-KOV_2_ (purple) incubations with human lung cancer (H460, H157, H838), human breast cancer (MCF-7), human prostate cancer (PC-3), human glioblastoma (U251MG), human microvascular endothelial (HMEC-1), and human lung fibroblast (MRC-5) cell lines. The tested cells were treated with different peptides for 24 h. Cells of positive and negative groups were treated with 0.1% Triton X-100 and serum free medium, respectively. All sets of experimental data represent the means ± SEMs of nine replications in three experiments and the significant differences were calculated by comparing various treatments with the negative control (**** *p* < 0.0001).

To explore the anticancer cell effect of kunitzin-OV_2_ and its analogues, six cancer cell lines were selected to determine their antiproliferative activity and selectivity (Figure 7). The results showed that kunitzin-OV_2_ and F9-KOV_2_ had no significant inhibitory effect on the tested cancer cell lines at their highest experimental concentrations. As expected, the conjugates, TAT-KOV_2_ and TAT-F9-KOV_2_, showed antiproliferative effects, especially TAT-F9-KOV_2_, which showed antiproliferative effects on the six cancer cell lines at different concentrations. Compared with TAT-KOV_2_, the antiproliferative effect of TAT-F9-KOV_2_ on H157, H838, H460, MCF-7, and U251MG cancer cells was significant even at the concentration of 10 µM, while at the same concentration, this mutant peptide did not inhibit the proliferation of normal human microvascular endothelial cells (HMEC-1) and pulmonary fibroblast cells (MRC-5). The IC_50_ values of TAT-KOV_2_ and TAT-F9-KOV_2_ are shown in Table 5. Among these, the antiproliferative effect of TAT-F9-KOV_2_ on the H157 cell line was the most potent, with an IC_50_ value of 2.2 µM. In addition, the antiproliferative effect of TAT-F9-KOV_2_ on PC-3 cancer cells was improved compared with TAT-KOV_2_.

#### 3.2.8. Haemolysis Effect on Horse Red Blood Cells

**Figure 8:**
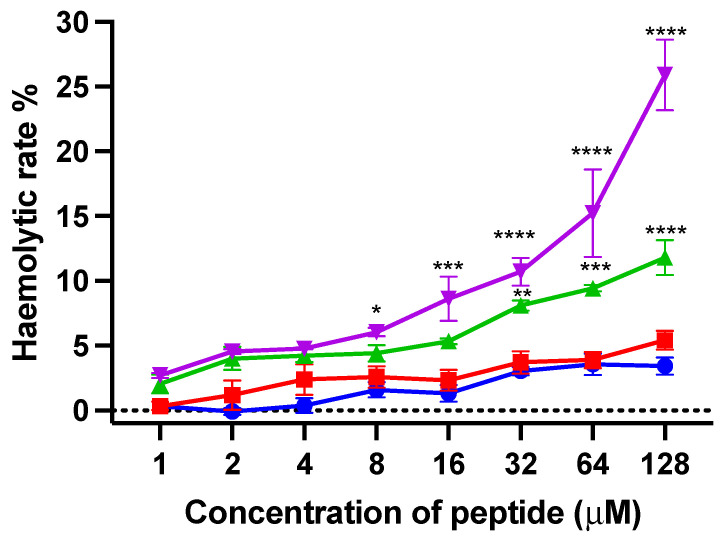
The haemolytic activity of kunitzin-OV_2_ (blue), F9-KOV_2_ (red), TAT-KOV_2_ (green), and TAT-F9-KOV_2_ (purple) at concentrations from 1 to 128 µM. Horse erythrocytes (2% in PBS) treated with 0.1% Triton X-100 and PBS were used as the positive and negative controls, respectively. The error bar represents the SEM of nine replications in three experiments and the significant differences were calculated by comparing various treatments with the negative control (* *p* < 0.05, ** *p* < 0.01, *** *p* < 0.001, and **** *p* < 0.0001).

Horse red blood cells were used to evaluate the haemolytic activity of kunitzin-OV_2_ and its analogues. As shown in Figure 8, the haemolytic activities of kunitzin-OV_2_ and F9-KOV_2_ were less than 10% even at the maximum test concentration (128 µM). For the conjugates, the haemolytic activity of TAT-KOV_2_ was not improved, only reaching 10% at its highest test concentration, while the haemolytic activity of TAT-F9-KOV_2_ was slightly improved compared with TAT-KOV_2_, reaching 10% at the concentration of 32 µM and about 25% at 128 µM. The results showed that only a small difference in haemolytic activity was observed between the natural peptide and the conjugates, indicating that the coupling of a cationic CPP would not cause strong erythrocyte cytotoxicity.

#### 3.2.9. LDH Cytotoxicity Assays

**Figure 9:**
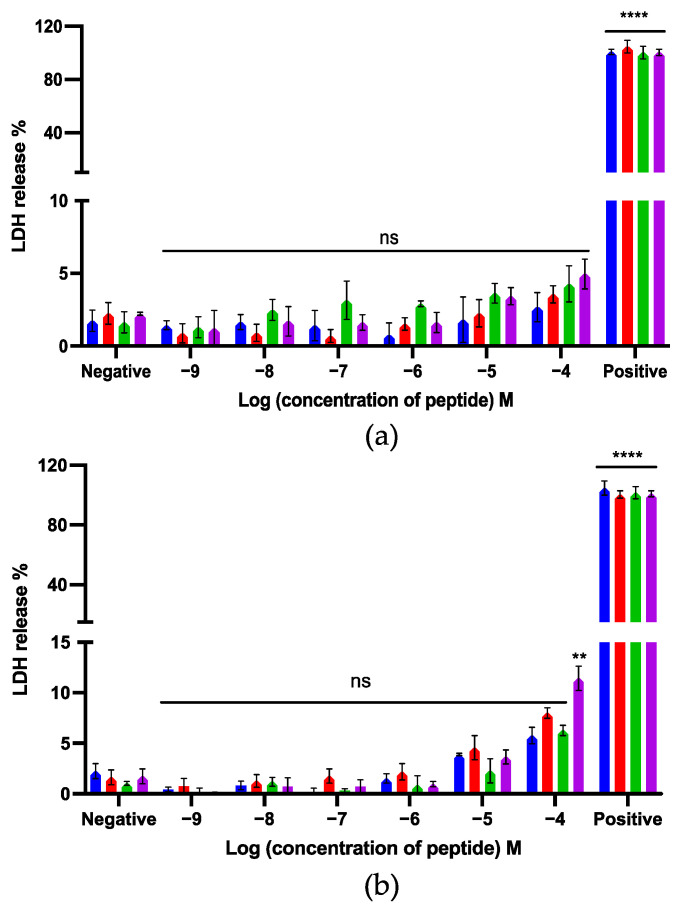
The LDH release of kunitzin-OV_2_ (blue), F9-KOV_2_ (red), TAT-KOV_2_ (green), and TAT-F9-KOV_2_ (purple) from (**a**) human microvascular endothelial (HMEC-1) and (**b**) human lung fibroblast (MRC-5) cell lines. HMEC-1 and MRC-5 cells were treated with different peptides for 24 h. Cells treated with 1 × lysis buffer and serum free medium were regarded as the positive and negative controls, respectively. The percentage of LDH release was calculated based on the maximum LDH release rate induced by the lysis buffer in the LDH experimental kit. The error bar represents the SEM of nine replications in three experiments and the significant differences were calculated by comparing different sample groups with the negative control (** *p* < 0.01, **** *p* < 0.0001, ns: No significance).

LDH assays were used to evaluate the cytotoxicity of kunitzin- OV_2_ and its analogues on MRC-5 and HMEC-1 cell lines. Compared with the positive control, kunitzin-OV_2_ and its analogues did not produce high LDH release from these two normal cell lines (Figure 9). Among these, only TAT-F9-KOV_2_ showed more than 10% LDH release at the highest test concentration of 100 µM against MRC-5 cells. It seems that conjugation with a cationic CPP can effectively preserve the low cytotoxicity of the parent peptide to MRC-5 and HMEC-1 cells, while exerting antibacterial and anticancer effects.

## 4. Discussion

Amphibian skin secretions contain a number of active substances, among which bioactive peptides have extensively been of great interest in natural drug development [30]. In recent years, several short KTIs have been found in amphibian skin secretions and these exhibit multiple biological functions with a substantial proportion exhibiting the bifunctionality of trypsin inhibition and antibacterial action. However, these KTIs cannot inhibit intracellular functions by disrupting cell membrane integrity, similar to most AMPs, due to their lower number of positive charges and lack of helical structures [10,11], etc.

In the present study, a novel KTI, named kunitzin-OV_2_, was discovered in the skin secretion of the Asian frog, *Odorrana versabilis.* This peptide was subsequently conjugated to the cationic CPPs, TAT, as well as its phenylalanine substituted analogue, in an attempt to improve their antibacterial and anticancer cell activities.

Similar to other reported amphibian-derived KTIs, kunitzin-OV_2_ exhibited a potent trypsin inhibitory function due to the basic amino acid, lysine, located at the P1 position, which produces a tighter binding to trypsin when interacting with the S1 pocket of the trypsin negatively-charged Asp189 [31]. However, in chymotrypsin, the S1 pocket is relatively non-polar, allowing binding only to aromatic or non-polar aliphatic amino acid residues [32], thus the substituted analogue F9-KOV_2_ exhibited clear chymotrypsin inhibitory activity when the lysine residue at the P1 position of the inhibitor was replaced with a phenylalanine residue. As expected, the conjugates of TAT with kunitzin-OV_2_ and F9-KOV_2_ retained their inhibitory function for trypsin and chymotrypsin, respectively. This agrees with a previous study of a TAT-BBI conjugate by Miao et al., fully illustrating that conjugating TAT to a trypsin inhibitor does not significantly alter its original protease inhibitory function [26].

To increase the bioactivity of certain AMPs, there has been some recent research describing the conjugation of cationic CPPs in an attempt to improve their antibacterial and anticancer activities by improving their cell penetration abilities [16,17,18]. Lee et al. indicated that the conjugation of arginine-rich, cell-penetrating peptide, R9, with classic AMPs (magainin and M15), significantly boosted their antibacterial activity against *E. coli* [33]. In terms of anticancer research, Chen et al. chose to add TAT to the N-terminus of the AMP, MP-C, boosting its anticancer cell proliferation ability more than 5-fold [34]. Moreover, past studies have shown that cationic CPPs alone did not exhibit clear antibacterial and anticancer effects, and the simultaneous introduction of CPPs with AMPs without undergoing coupling did not achieve synergistic effects on antibacterial activity [26,33]. Therefore, the conjugation with cationic CPPs can be an essential factor to increase the biological activities of bioactive peptides.

In the study of antibacterial activity, kunitzin-OV_2_ exhibited the ability to inhibit and kill *E. coli* with an MIC of 128 µM, while for F9-KOV_2_, its antibacterial ability at the highest experimental concentration was lost, probably due to kunitzin-OV_2_ bearing one more positive charge than F9-KOV_2_, making it more electrostatically attractive to negatively-charged bacterial cell membranes. Next, TAT was conjugated to the natural peptide in an attempt to increase the antibacterial activity and, as expected, TAT-KOV_2_ and TAT-F9-KOV_2_ exhibited a significant increase in antibacterial effect against *E. coli*, which was actually better than *S. aureus*. Previous studies showed that the surface of *E. coli* carries more negative charges than *S. aureus*, which is associated with negatively-charged lipopolysaccharides (LPS) on the outer protective membrane of this Gram-negative bacteria [35,36]. Conjugation of cationic CPPs adds a large number of positive charges to the natural peptide complex, thus increasing its binding capacity to the LPS of Gram-negative bacteria [33]. However, these conjugates only produced weak antibacterial effects against other tested Gram-negative bacteria (e.g., *P. aeruginosa* and *K. pneumoniae*) at the highest tested concentration and the parent peptides as well as the conjugates seemed to possess a high specificity for *E. coli*. A possible reason for this was that *P. aeruginosa* and *K. pneumoniae* were more likely to cause resistance to the peptide than *E. coli*. These Gram-negative pathogens use many different mechanisms to resist antibacterial effects of cationic peptides, which include altering their own surface structure to repel or diminish peptide binding using efflux pumps to expel and sequester peptides or producing proteases for peptide degradation purposes [37,38]. Furthermore, in the membrane permeability experiments, these tested peptides did not produce additional membrane lysis, which illustrates that, unlike the conventional membrane disruption bactericidal mechanism of AMPs [39], the parent peptides and the conjugates appear to target intracellular targets in bacteria in a non-membrane destructive manner. This is in accordance with previous studies which found that some AMPs with protease inhibitory function can inhibit proteases within bacterial cells, for example, ixodidin, an AMP isolated from the blood cells of *Boophilus microplus* which has a protease inhibitory function, can target the bacterial intracellular serine proteases and exert antibacterial effects against *E. coli* by inducing the cessation of cellular metabolism [40]. These studies can further support the speculation about the exact antibacterial mechanism of kunitzin-OV_2_ and its derivatives. However, the specific mechanism of action of these tested peptides and the reasons for producing specific antibacterial activity against *E. coli* are still unclear and require further experimental investigation. Subsequently, we compared the antibacterial activity of kunitzin-OV_2_ (MIC/MBC = 128 µM (182.1 μg/mL)), TAT-KOV_2_ (MIC/MBC = 4 µM (11.4 μg/mL)), and TAT-F9-KOV_2_ (MIC/MBC = 8 µM (23.0 μg/mL)) with the typical AMP melittin (MIC/MBC = 40–42.5/64–128 μg/mL) and found that the conjugates were 2–4-fold more effective against *E. coli* than melittin, along with a 3–10-fold increase in bactericidal ability [41]. Combined with the results of the low cytotoxicity of the conjugates, this mode of conjugation of TAT to low toxicity peptides may also effectively avoid the drawbacks of the high cytotoxicity of traditional AMPs. Moreover, TAT-peptide conjugates exhibited better salt stability than kunitzin-OV_2_, which was beneficial to guarantee their antibacterial effects under multiple physiological contexts. Surprisingly, in the following time-killing kinetic experiments, it was found that TAT conjugation accelerated the bactericidal rate of *E. coli* at its MIC compared with kunitzin-OV_2_, by 30 min, indicating that the cationic CPP could effectively improve the peptide’s bactericidal rate under the conditions of low effective bactericidal concentration.

In addition to these experiments, those which analyzed peptide performance against the generation and eradication of biofilm of *E. coli*, showed that the peptides did not have an inhibitory effect on the biofilms that had been generated, regardless of conjugation with TAT, but had an inhibitory effect on biofilm formation at concentrations of conjugates exceeding 2-fold MICs. Combined with the results of time-killing experiments, it appears that the biofilms formed before the bacteria were completely killed, which resulted in the inability of both the parent peptide and its conjugate to inhibit biofilm formation at lower concentrations, whereas these peptides were no longer inhibitory for the already generated biofilms [28,42].

In anticancer research, protease inhibitors have played a significant role [43,44]. To date, the anticancer ability of KTIs extracted from amphibian skin secretions has not been investigated. In a previous study, Zhang et al. found that the BBI type chymotrypsin inhibitor, HECI, found in *Hylarana erythraea*, demonstrated potent inhibition of proliferation in non-small cell lung cancer cell line, H157 [45]. In this study, kunitzin-OV_2_ and F9-KOV_2_ did not exert antiproliferative effects at the highest tested concentrations. In general, anticancer peptides exert their antiproliferation activity by binding to negatively-charged phosphatidylserine exposed on the cancer cell surfaces [46,47], while kunitzin-OV_2_ and F9-KOV_2_, which lack positive charges and helical structures, cannot affect cancer cell membrane internalization through binding to phosphatidylserine, thus they lack the ability to translocate into cancer cells at the experimental concentrations used. As expected, the conjugate TAT-KOV_2_ exhibited a significant boost in antiproliferative effects against cancer cells. However, TAT-F9-KOV_2_ showed better anticancer cell activity than TAT-KOV_2_, in which TAT-F9-KOV_2_ increased the antiproliferation effects on H157, H838, and H460 cells by 10–20-fold and inhibited U251MG and MCF-7 cells at a concentration of 10 µM. The reason that these two conjugates had different anticancer cell activities is related to the anticancer cell effects exerted by the protease inhibitors and are generated through inhibition of the different subunits of the intracellular 20 S proteasome [45,48,49]. The 20 S proteasome, as the core particle of the human 26 S proteasome, has three major active subunits, (caspase-like) β1, (trypsin-like) β2, and (chymotrypsin-like) β5, and the inhibition of these three subunits plays an important role in mediating the apoptotic process, which has been confirmed in previous research [45,46,47,48,49,50,51,52]. Here, it is speculated that the chymotrypsin inhibitor has a higher affinity for the 20 S proteasome in the tested cancer cell lines [45,48,52], especially in H157, H460, U251MG, and MCF-7 cells, compared with the trypsin inhibitor. This assertion would require further 20 S proteasome inhibition experiments for confirmation. The utilization of cationic CPPs for coupling to peptides with chymotrypsin inhibitor function provides a new insight for the development of anticancer agents in the future.

Furthermore, the conjugates seem to be somewhat targeted to cancer cells. The peptides analyzed in this study did not induce haemolysis toward horse red blood cells and did not cause significant LDH leakage in HMEC-1 cells or in MRC-5 cells at the highest antiproliferative concentrations used. However, it is worth noting that the possible nephrotoxicity caused by arginine-rich CPPs has been identified as a problem [53,54,55]. Moulton et al. found that in the study of the conjugate (PPMO) of phosphodiamidate morpholino oligomers (PMO) and cationic CPP in various animal models of duchenne muscular dystrophy (DMD), although cationic CPP could effectively improve the uptake of PMO by cells, the animals treated with high-dose PPMO suffered severe renal failure, and the toxicity level was related to animal species in which monkeys were more sensitive to the toxicity of the cationic CPP conjugates than mice [54]. In addition, Amantana et al. found that CPP-PMO accumulated in the kidney and liver, which was higher than its content in other tissues and the toxicity of CPP-PMO showed a dose-dependent manner [53]. These experiments clearly showed that the conjugation of cationic CPP is an effective way to enhance the internalization of cargos, but the way to improve the safety of conjugates needs further research.

## 5. Conclusions

In summary, kunitzin-OV_2_ is a novel peptide discovered in frog skin secretion and displays trypsin inhibition and weak antibacterial activity. This is the first study to describe the conjugation of a CPP with an amphibian-derived KTI. The results showed that conjugates formed with TAT and natural or analogue peptides with different protease inhibitory functions did not exhibit strong cytotoxicity, but showed a significant enhancement of antibacterial as well as anticancer cell activities. Therefore, TAT-KOV_2_ and TAT-F9-KOV_2_ are synthetic peptides which have the potential to be dual biofunctional agents and provide new insights for the manipulation and development of natural drugs in the future.

## Figures and Tables

**Table 1:** The sequences and physicochemical properties of kunitzin-OV_2_ and its rational derivatives.

Peptide Name	Sequence	Net Charge	GRAVY ^a^
Kunitzin-OV_2_	AAKLPFRCKAAFC	+3	0.592
F9-KOV_2_	AAKLPFRCFAAFC	+2	1.108
TAT-KOV_2_	RKKRRQRRRGGAAKLPFRCKAAFC	+11	−1.308
TAT-F9-KOV_2_	RKKRRQRRRGGAAKLPFRCFAAFC	+10	−1.029

^a^ GRAVY—the grand average of hydropathicity. +GRAVY values indicate hydrophobic; −GRAVY values indicate hydrophilic.

**Table 2:** The Ki value of kunitzin-OV_2_, F9-KOV_2_, TAT-KOV_2_, and TAT-F9-KOV_2_ against trypsin and chymotrypsin. “N.I.” indicates no inhibitory effect.

Peptide	Sequence	Trypsin Ki (µM)	Chymotrypsin Ki (µM)
Kunitzin-OV_2_	AAKLPFRCKAAFC	9.82	N.I.
F9-KOV_2_	AAKLPFRCFAAFC	N.I.	2.19
TAT-KOV_2_	RKKRRQRRRGGAAKLPFRCKAAFC	10.11	N.I.
TAT-F9-KOV_2_	RKKRRQRRRGGAAKLPFRCFAAFC	N.I.	2.23

**Table 3:** MIC/MBC of kunitzin-OV_2_ and its relatives against different microorganisms.

MIC/MBC (µM)						
Microorganism		Kunitzin-OV_2_	F9-KOV_2_	TAT-KOV_2_	TAT-F9-KOV_2_	TAT ^a^
Gram-positive	*S. aureus* (ATCC 6538)	>128	>128	32/64	64/128	>128
	MRSA (NCTC 12493)	>128	>128	>128	>128	>128
	*E. faecalis* (NCTC 12697)	>128	>128	128/>128	>128	N.A.
Gram-negative	*E. coli* (ATCC 8739)	128/128	>128	4/4	8/8	>128
	*P. aeruginosa* (ATCC 9027)	>128	>128	>128	>128	>128
	*K. pneumoniae* (ATCC 43816)	>128	>128	>128	>128	N.A.
Yeast	*C. albicans* (ATCC 10231)	>128	>128	>128	>128	>128

^a^ Data from [26]; “N.A.” indicates data not applicable.

**Table 4:** The MICs (μM) of kunitzin-OV_2_, F9-KOV_2_, TAT-KOV_2_, and TAT-F9-KOV_2_ against *E. coli* in the presence of different salts.

MIC (µM)								
Peptide	PBS	NaCl	KCl	NH_4_Cl	ZnCl_2_	MgCl_2_	CaCl_2_	FeCl_3_
Kunitzin-OV_2_	128	>128	>128	>128	>128	>128	>128	>128
F9-KOV_2_	>128	>128	>128	>128	>128	>128	>128	>128
TAT-KOV_2_	4	4	4	4	4	8	16	4
TAT-F9-KOV_2_	8	8	8	8	8	16	32	8

**Table 5:** The IC_50_ value (μM) of TAT-KOV_2_ and TAT-F9-KOV_2_ against tested cell lines after 24 h of treatment.

Peptide	H157	H838	H460	U251MG	MCF-7	PC-3	HMEC-1	MRC-5
TAT-KOV_2_	45.8	92.4	49.2	N.I.	N.I.	96.8	166.4	64.9
TAT-F9-KOV_2_	2.2	9.9	5.1	7.6	11.7	44.3	98.1	43.6

## Data Availability

The mature peptide identified from the skin secretion was named kunitzin-OV_2_, and the nucleotide sequence of cDNA has been deposited in the GenBank database under accession number ON866881.

## Supplementary Materials

The following supporting information can be downloaded at: https://www.mdpi.com/article/10.3390/pharmaceutics14091805/s1. Figure S1: MALDI-TOF mass spectra of the purified peptides (A) kunitzin-OV_2_, (B) F9-KOV_2_, (C) TAT-KOV_2_, (D) TAT-F9-KOV_2_; Figure S2: RP-HPLC chromatograms of purified peptides (A) kunitzin-OV_2_, (B) F9-KOV_2_, (C) TAT-KOV_2_, (D) TAT-F9-KOV_2_.
