# Supplementary material for: Conjugation of a Cationic Cell-Penetrating Peptide with a Novel Kunitzin-like Trypsin Inhibitor: New Insights for Enhancement of Peptide Bioactivities
Source: Pharmaceutics. 2022 Aug 27;14(9):1805. doi: 10.3390/pharmaceutics14091805 (PMC9501525; doi:10.3390/pharmaceutics14091805)
Supplement: Supplementary file 1 [file pharmaceutics-14-01805-s001.zip › pharmaceutics-1825361-supplementary.pdf]

Article

Conjugation of a Cationic Cell-Penetrating Peptide with a Novel Kunitzin-Like Trypsin Inhibitor: New Insights for Enhancement of Peptide Bioactivities

Junting Yao [1,†], Weining Yin [1,†], Yuqing Chen [1,2,†], Xiaoling Chen [1,*], Yangyang Jiang [1], Tao Wang [1], Chengbang Ma [1], Mei Zhou [1], Tianbao Chen [1], Chris Shaw [1] and Lei Wang [1,*]

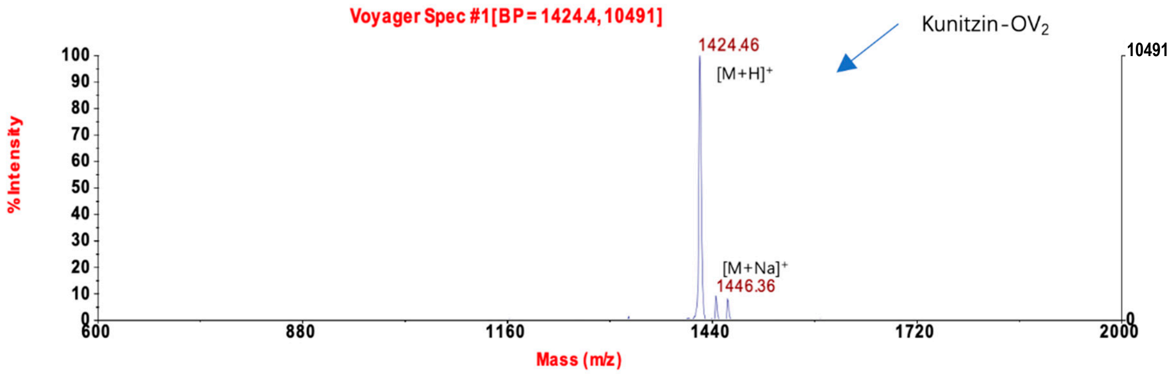

(A)

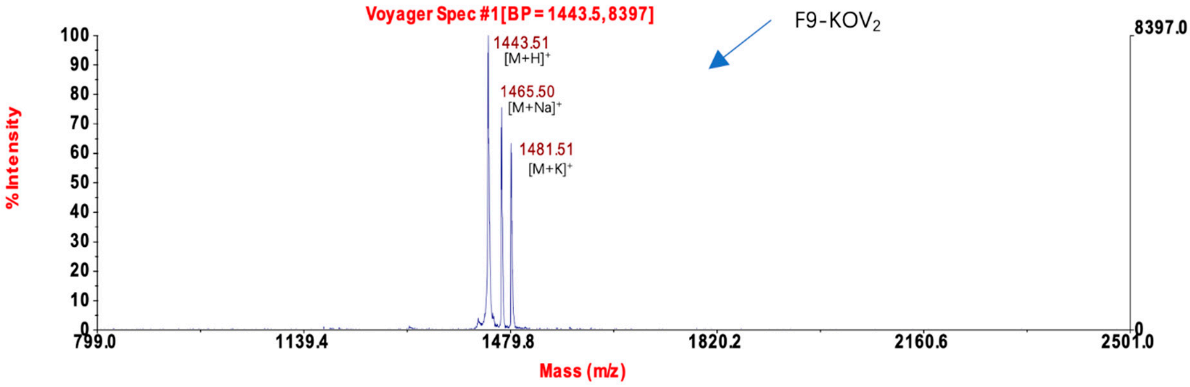

(B)

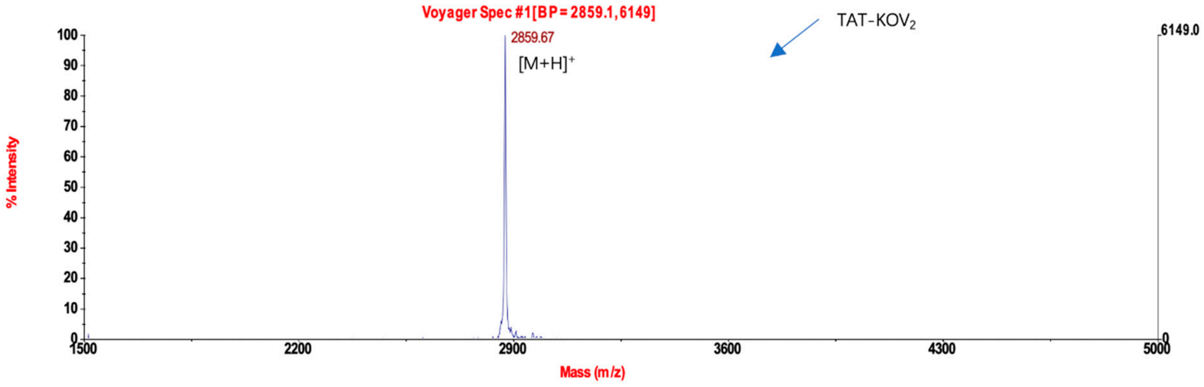

(C)

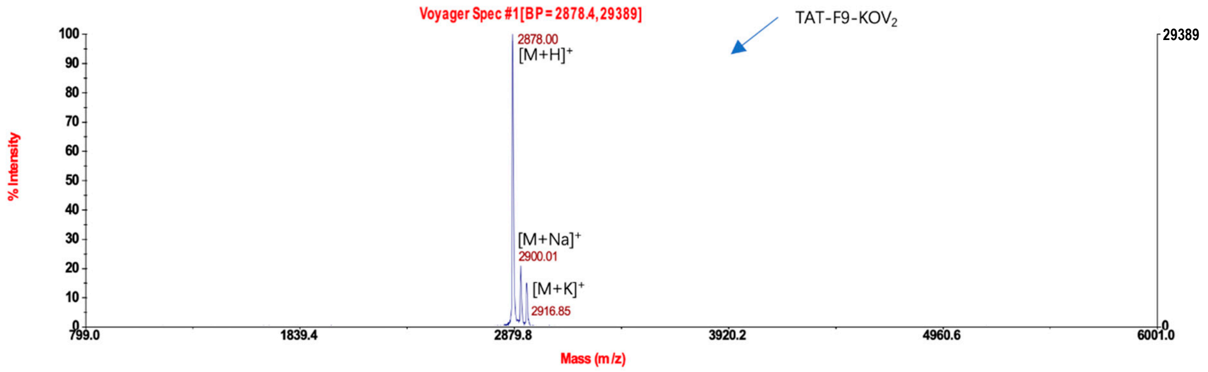

(D)

Figure S1: MALDI-TOF mass spectra of the purified peptides (A) kunitzin-OV_2, (B) F9-KOV_2, (C) TAT-KOV_2, (D) TAT-F9-KOV_2. The elution peak of each peptide is indicated by an arrow.

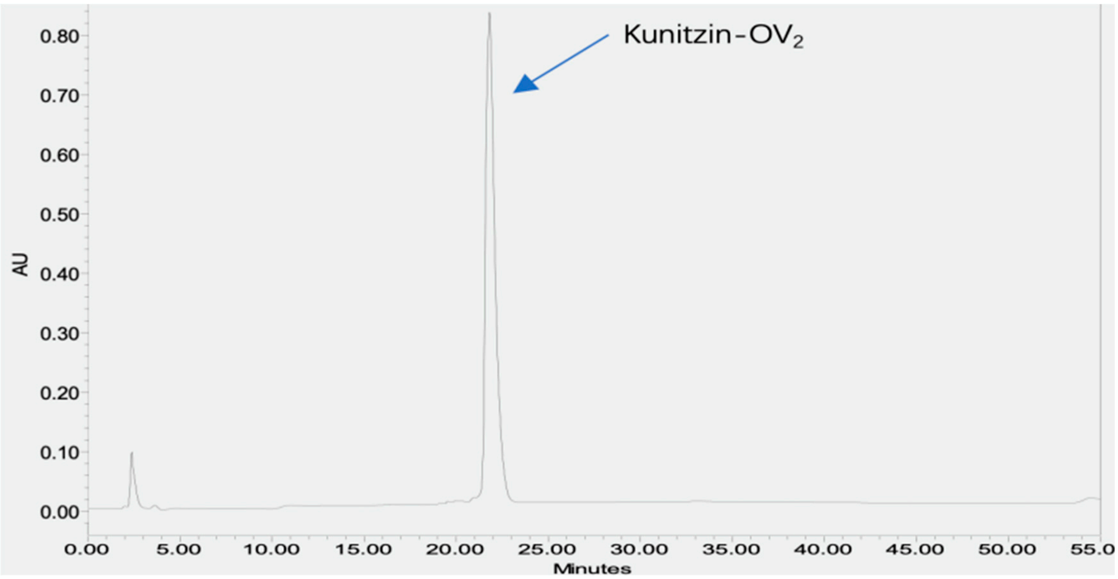
Kunitzin-OV2
AU
0.00
0.10
0.20
0.30
0.40
0.50
0.60
0.70
0.80
0.00 5.00 10.00 15.00 20.00 25.00 30.00 35.00 40.00 45.00 50.00 55.0
Minutes

(A)

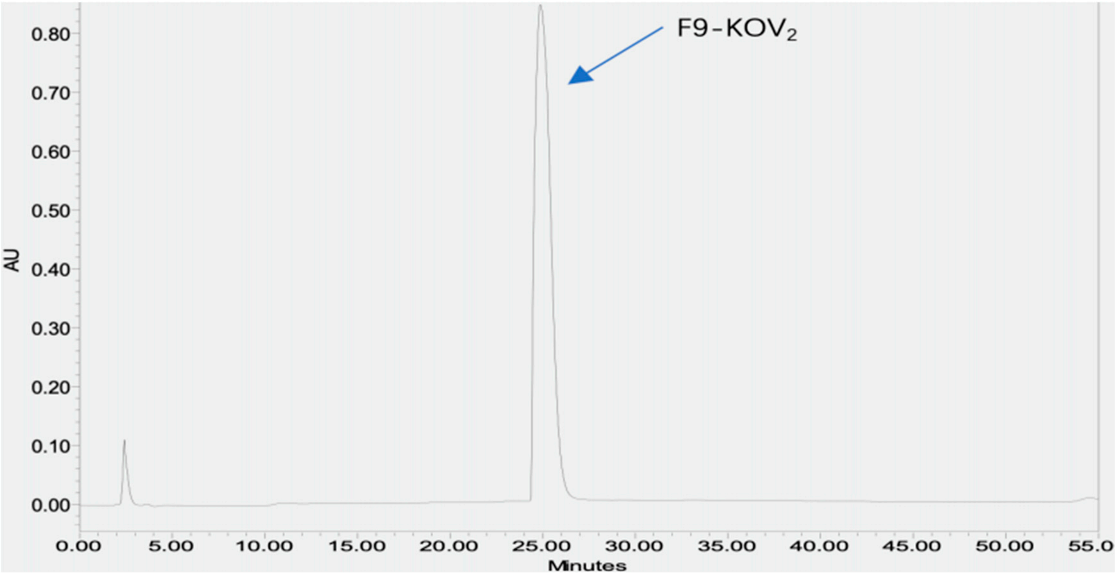
F9-KOV2
AU
0.00
0.10
0.20
0.30
0.40
0.50
0.60
0.70
0.80
0.00 5.00 10.00 15.00 20.00 25.00 30.00 35.00 40.00 45.00 50.00 55.0
Minutes

(B)

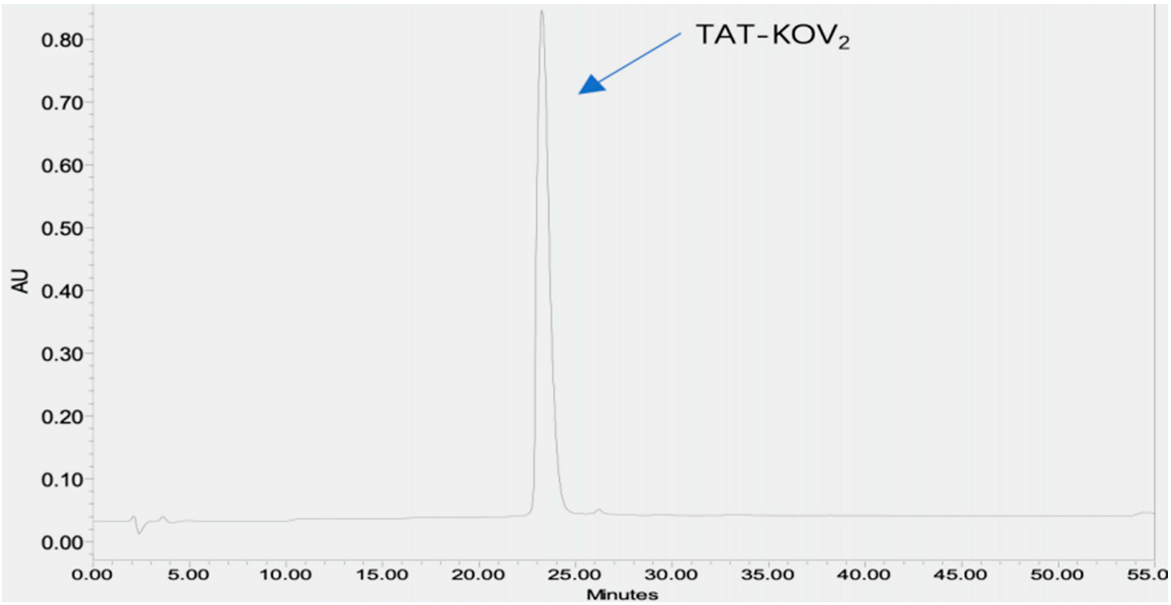

(C)

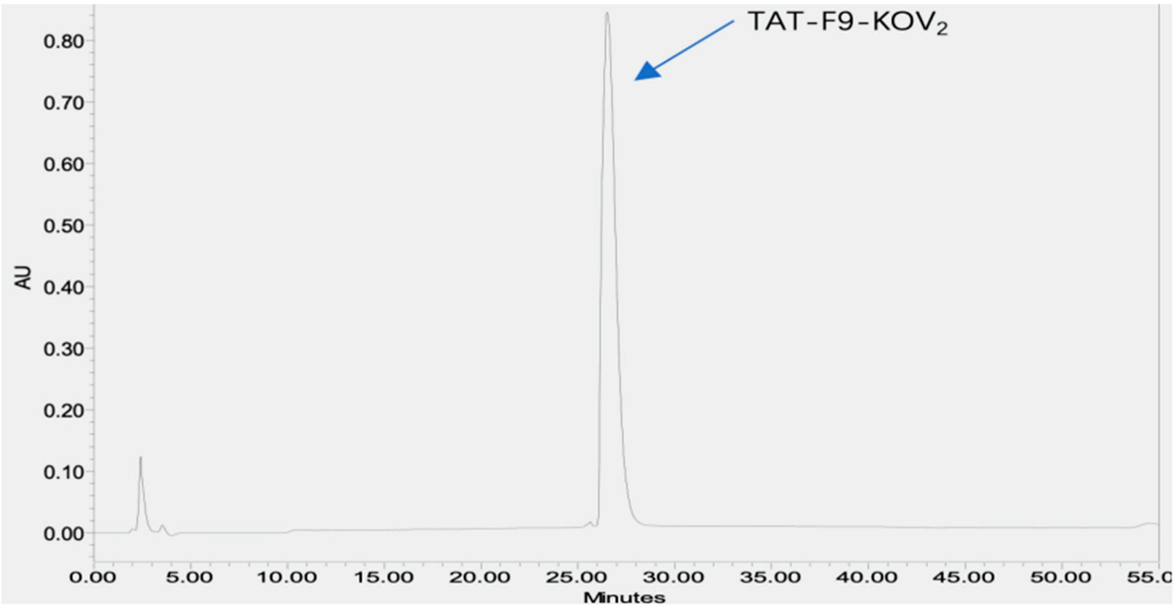

(D)

Figure S2: RP-HPLC chromatograms of purified peptides (A) kunitzin-OV_2, (B) F9-KOV_2, (C) TAT-KOV_2, (D) TAT-F9-KOV_2. The elution peak of each peptide is indicated by an arrow.
